# Evaluation of the Usefulness of the MRI Jelly Method for Diagnosing Complete Cul-de-Sac Obliteration

**DOI:** 10.1155/2014/437962

**Published:** 2014-04-10

**Authors:** Iwaho Kikuchi, Ryohei Kuwatsuru, Kana Yamazaki, Jun Kumakiri, Yoichi Aoki, Satoru Takeda

**Affiliations:** ^1^Department of Obstetrics and Gynecology, Juntendo University Faculty of Medicine, Hongo 2-1-1, Bunkyo-ku, Tokyo 113-8421, Japan; ^2^Department of Radiology, Juntendo University Faculty of Medicine, Hongo 2-1-1, Bunkyo-ku, Tokyo 113-8421, Japan

## Abstract

*Objective.* We conducted a single-center study to evaluate the usefulness of the magnetic resonance (MR) imaging jelly method for diagnosing endometriosis-associated adhesions in the Pouch of Douglas. *Methods.* Thirty women with menstrual pain, dyspareunia, and chronic pelvic pain were enrolled in the study. All had been scheduled for laparoscopic surgery on the basis of pelvic and/or ultrasonographic (US) evaluation. All underwent MR imaging both with and without application of US jelly to the vagina and rectum. The images were compared and analyzed postsurgically in a random and blinded fashion by a radiology specialist and a radiology fellow. The radiologists' interpretations of the images were compared to the surgical findings recorded on DVDs. *Results.* Adhesions in the Pouch of Douglas were found in 21 patients. The sensitivity and specificity of MR imaging without jelly administration were 85.7% and 55.6%, respectively, for the specialist and 81.0% and 55.6%, respectively, for the fellow; with jelly administration, values were 95.2% and 88.9% for the specialist and 90.5% and 66.7% for the fellow. Opacity produced by the jelly increased the sensitivity and specificity for both radiologists. *Conclusion.* The MRI jelly method is a potentially useful, beneficial, and simple approach for diagnosing Pouch of Douglas adhesions.

## 1. Introduction

Endometriosis is roughly classified into three types: peritoneal endometriosis, ovarian chocolate cyst, and rectovaginal endometriosis [1]. Rectovaginal endometriosis in particular can cause the most severe symptoms including dysmenorrhea, dyspareunia, defecation pain, and chronic pelvic pain, all of which can compromise the patient's quality of life (QOL) [2]. Deep infiltrating endometriosis (DIE) is a particularly severe type of rectovaginal endometriosis. In the most severe cases, DIE results in complete cul-de-sac obliteration (CCDSO), which is evident upon laparoscopic examination [3, 4].

Adhesiolysis for CCDSO is technically demanding, and the incidence of complications is higher than when routine gynecologic laparoscopy procedures are performed because the Pouch of Douglas is adjacent to critical organs including the rectum and ureters [4]. Thus, presurgical diagnosis is very important for preoperative planning and for obtaining meaningful informed consent. However, a standard diagnostic imaging method has not yet been established for DIE.

The magnetic resonance (MR) imaging jelly method can be used to delineate the locational relationships between the uterus, vagina, and rectum and to assess adhesions in the Pouch of Douglas. The method involves application of ultrasonography (US) jelly in the vagina and rectum during MR imaging to enhance contrast [5, 6]. US jelly is characteristically hypointense on T1-weighted images and hyperintense on T2-weighted images. In the present study, we aimed to evaluate the usefulness of the MR imaging jelly method in the diagnosis of adhesions in the Pouch of Douglas.

## 2. Materials and Methods

### 2.1. Study Patients

Thirty women were enrolled in the study. Assuming a true diagnostic rate of 90% for identification of DIE on MR images obtained with jelly and a true diagnostic rate of 50% for identification of DIE on MR images obtained without jelly, we determined that a sample size of 19 patients would be needed to yield a statistical power of 80%. Taking a certain safety margin into account, a sample of 30 women was specified in the study protocol. Outpatients were recruited through our Juntendo University Hospital Department of Obstetrics and Gynecology between April 2010 and March 2012. Women presenting with menstrual pain, dyspareunia, and chronic pelvic pain were eligible for the study (1) if they received a preoperative diagnosis of benign gynecologic disease, that is, myoma, adenomyosis, and/or endometriosis by pelvic examination or by US, (2) if the diagnosed condition was indicated for laparoscopic surgery, (3) if they were scheduled to undergo such surgery at our hospital, and (4) if they provided written informed consent to participate in the study as outlined in the study protocol, which involved undergoing MR imaging twice (before and after administration of the jelly). The study protocol, including the MR imaging jelly method, was explained in writing to patients, who also provided informed consent for their imaging data and operative findings to be used for the study.

### 2.2. MR Imaging, with and without Jelly

We worked together with our hospital's radiology department to schedule specific times during which our study patients could be admitted for imaging. MR imaging was performed on a 1.5T scanner (VISART EX, Toshiba; Nasu, Japan) equipped with a phased array body coil to obtain T1- and T2-weighted images. Axial and sagittal T2-weighted fast spin echo images and sagittal T1-weighted spin echo images with fat saturation were obtained as 5-6 mm thick contiguous slices. All images were obtained with a 25–27 cm × 25–27 cm field of view and a 256 × 256 matrix. All images were stored in our hospital's picture archiving and communication system (PACS).

A gynecologist, one of the four gynecologists in the team of investigators, was present at each of the imaging sessions and applied the US jelly to each of the patients. Each patient was given a senna extract (0.5 g of Alosenn) to be taken at bedtime for 3 consecutive days before their scheduled exam. This was done to empty the rectum. Pelvic MR imaging was first performed routinely as described above. Then, with the patient lying on her side on the examination table in the MR imaging room, US jelly (Echo Jelly, Hitachi Aloka Medical, Ltd., Tokyo, Japan) was introduced by the gynecologist into the patient's vagina and rectum by means of our own customized 16 French Nelaton catheter attached to a single syringe. The vagina was filled with 50 mL of the jelly, and the rectum was then filled with 150 mL of the jelly diluted twice with tap water. After completion of the imaging, the patient returned home without undergoing any other procedure. The water soluble jelly was easily washed out at home during bathing.

### 2.3. Surgical Procedure

All 30 patients underwent laparoscopic surgery under general anesthesia with endotracheal intubation. Patients were placed in the lithotomy position, a Veress needle was inserted through the umbilical region into the peritoneal cavity, and carbon dioxide gas was used to create pneumoperitoneum. Four trocars were inserted: a 10 mm trocar for the scope at the umbilical region; two 5 mm trocars, one on either side of the iliac spine; and one 10 mm trocar in the anterior axillary line, slightly above the umbilical region.

The uterus was anteverted with the use of a Uterine Manipulator (Atom Medical Corp., Tokyo, Japan), and the posterior vaginal fornix was pushed cephalad with the tip of the shaft of the manipulator. The presence or type of CCDSO was determined according to the revised American Society for Reproductive Medicine (rASRM) classification [7]. That is, the cul-de-sac was judged to be normal when the bulge of the posterior vaginal vault was seen between the two uterosacral ligaments, partial cul-de-sac obliteration (PCDSO) was diagnosed when only part of the bulge of the posterior fornix bulge was seen, and CCDSO was diagnosed when the posterior vaginal vault could not be seen at all.

If CCDSO was diagnosed, the uterus was anteverted with a uterine manipulator, and the anterior wall of the rectum adhering to the posterior wall of the uterus was drawn cephalad with grasping forceps. The interface was opened with a monopolar needle and dissected bluntly, and the incision was repeated until the cul-de-sac was opened so that the uterosacral ligament and bulge of the posterior fornix were clearly seen.

### 2.4. Image Analysis

Patients' MR images were analyzed before the laparoscopic surgery was performed, and images obtained by the jelly method were used for final preoperative diagnosis. The images were interpreted with reference to six findings previously reported to be useful [6]: (1) uterine position (anteflexion or retroflexion: a retroflexed uterus was considered positive for CCDSO), (2) thickness of the posterior uterine wall (adenomyosis uteri: thickness of the muscular layer from the junctional zone ≥12 mm was considered positive), (3) ascites in the Pouch of Douglas (no ascites or ascites not reaching the level of the posterior vaginal vault was considered positive), (4) apparent tethering of the posterior vaginal vault (a beak-shaped posterior vaginal vault without a round bulge on MR images obtained with jelly was considered positive), (5) apparent tethering of the anterior wall of the rectum (a serrated anterior rectal wall without a smooth surface on MR images obtained with jelly was considered positive), and (6) a Pouch of Douglas lesion visualized as a high-intensity area on a T1-weighted image (T1WI) (a hyperintense lesion in the Pouch of Douglas between the hypointense vagina and rectum on a T1WI was considered positive). These findings were assessed within the overall context, and each radiologist made a final determination of whether CCDSO was present. Moreover, the radiologists reviewed anatomical abnormalities and/or deformities to the extent possible in making their final assessment of the presence or absence of CCDSO.

### 2.5. Comparisons

For the purpose of the study, MR images obtained with and without jelly (60 images in total) were extracted from the PACS and reanalyzed. The images were randomly extracted with the patients' names concealed. Furthermore, the images obtained with and without jelly were analyzed in random order.

The MR images were interpreted by the two aforementioned radiologists. One of these radiologists is a specialist who, at the time of the study, had 31 years of experience in imaging diagnosis, and the other is a fellow who, at the time of the study, had 2 years of experience in imaging diagnosis and had completed a 2-year residency. They independently analyzed the randomly extracted images and recorded their findings on a report sheet. The 6 findings described above and the final imaging diagnosis, that is, the presence or absence of CCDSO, were recorded on the sheet. A gynecologist (the first author) reviewed DVD recordings of the patients' laparoscopic surgeries and confirmed the presence or absence of CCDSO by checking the video findings against the surgical records. The presence of CCDSO was determined by this investigator according to the rASRM classification [7]. Specifically, CCDSO depended on the visible extent of the posterior vaginal vault during the adhesiolysis surgery recorded on DVD.

### 2.6. Statistical Analysis

Four imaging-based diagnoses were obtained for each patient because MR images before and after opacification with jelly in the same patient were interpreted by two readers. Diagnostic sensitivity, specificity, positive predictive value (PPV), and negative predictive value (NPV) were calculated for each of the imaging methods as interpreted by each of the two radiologists. We compared the diagnostic accuracies of the two imaging methods by examining the difference in areas under the receiver-operating characteristic (ROC) curves drawn for each method. The ROC curves were drawn to show the trade-off between sensitivity and specificity and thus reflected the accuracy of the respective imaging methods. All statistical analyses were performed with SPSS statistical software, version 18 (IBM, Tokyo, Japan).

## 3. Results

The 30 enrolled patients ranged in age from 23 to 45 years (mean, 36.4 ± 8.0 years). MR imaging examinations were performed for all 30 patients according to the above-described protocol. There were no adverse effects related to the MR imaging procedures, jelly administration, or surgery. Eleven of the 30 patients were diagnosed as having myoma/adenomyosis, four of whom underwent myomectomy and seven of whom underwent hysterectomy. Seventeen patients were diagnosed as having ovarian cyst(s) and underwent cystectomy. Two patients were found to have bowel endometriosis and underwent bowel resection. Of the total 30 patients, 21 had CCDSO. All 21 patients underwent complete adhesiolysis. The mean rASRM score was 68.6 ± 43.3 (range, 0–128).

The sensitivity, specificity, PPV, and NPV of MR imaging before jelly administration in the diagnosis of CCDSO, as interpreted by the radiology specialist, were 85.7% (18/21), 55.6% (5/9), 81.8% (18/22), and 62.5% (5/8), respectively. The sensitivity, specificity, PPV, and NPV of MR imaging before jelly administration in the diagnosis of CCDSO as interpreted by the radiology fellow were 81.0% (17/21), 55.6% (5/9), 81.0% (17/21), and 55.6% (5/9), respectively. Values for MR imaging with jelly administration in the diagnosis of CCDSO as interpreted by the radiology specialist were 95.2% (20/21), 88.9% (8/9), 95.2% (20/21), and 88.9% (8/9), respectively, and by the radiology fellow, they were 90.5% (19/21), 66.7% (6/9), 86.4% (19/22), and 75.0% (6/8), respectively.

**Figure 1:**
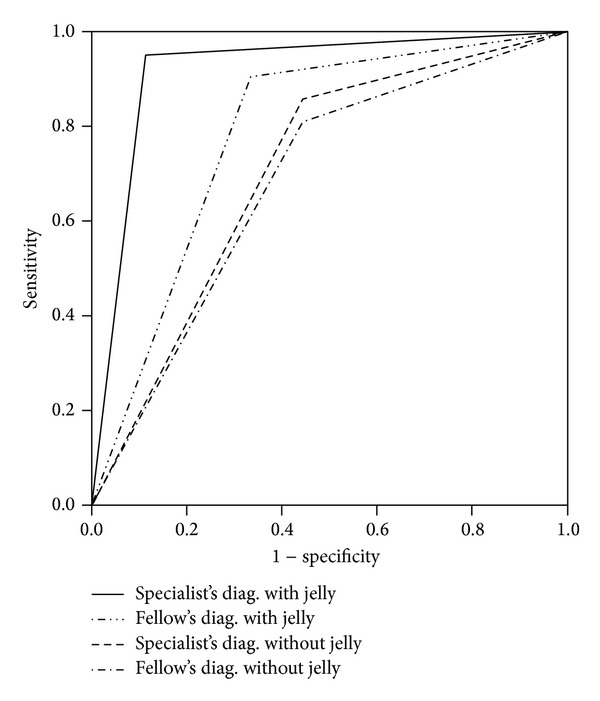
ROC curves. Greater sensitivity for a diagnosis of adhesions in the Pouch of Douglas was achieved for the specialist by MR imaging with jelly than by MR imaging without jelly. Greater sensitivity and specificity were achieved for the fellow by MR imaging with jelly.

The ROC curves are presented in Figure 1. For the specialist, statistically significant results (*P* = 0.001) were achieved without jelly. However, with jelly, greater sensitivity was achieved. For the fellow, greater sensitivity and specificity were achieved with jelly than without jelly.

### 3.1. Example MR Images

#### 3.1.1. Absence of CCDSO

**Figure 2:**
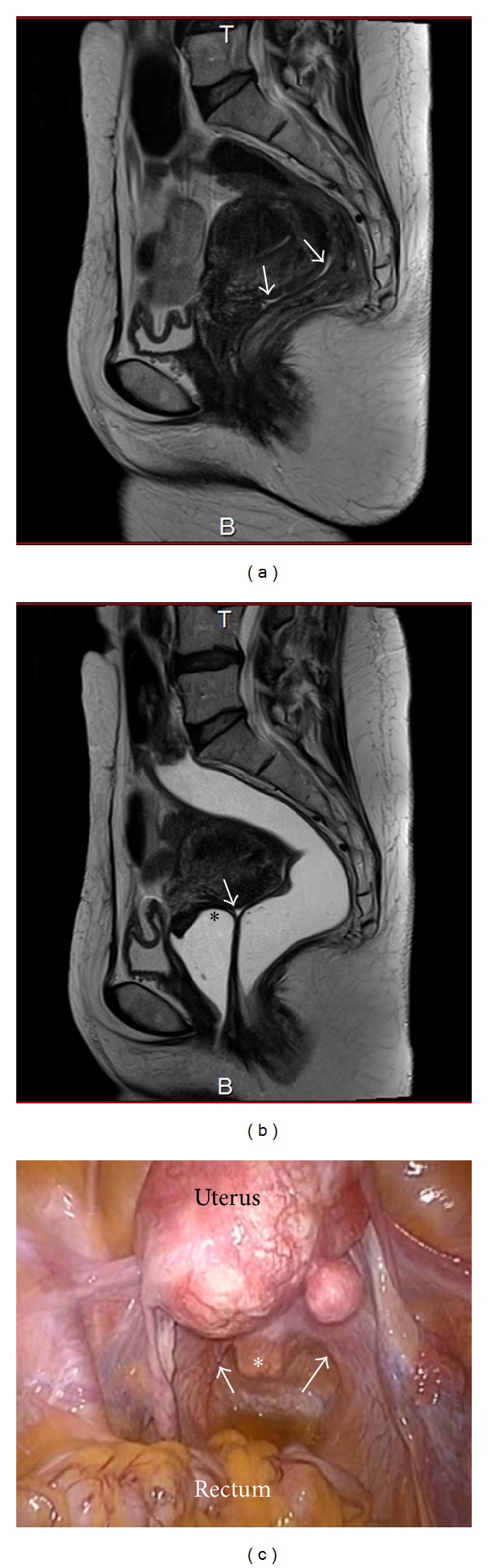
(a) MR image obtained before jelly administration in a patient with endometriosis but no CCDSO. Ascites is seen in the Pouch of Douglas (white arrow). (b) MR image obtained after jelly administration in the same patient. The surface of the rectal wall has a smooth appearance, and the posterior vaginal vault (asterisk) is visualized along with the ascites in the Pouch of Douglas (white arrow). (c) Laparoscopic findings in the same patient. The posterior vaginal vault is easily visualized in the area where the handle of the manipulator (asterisk) is seen. The uterosacral ligaments are also apparent (white arrow).

Images from a case of endometriosis without CCDSO are shown in Figures 2(a)–2(c).  Figure 2(a) is an MR image obtained before jelly administration. Ascites is present in the Pouch of Douglas, but no anatomical abnormality is present.  Figure 2(b) is an MR image obtained after jelly administration in the same patient. The surface of the rectal wall has a smooth appearance, and the posterior vaginal vault is visualized along with the ascites in the Pouch of Douglas. The image is interpreted as showing the absence of CCDSO.  Figure 2(c) is an image obtained upon laparoscopy in the same patient. The posterior vaginal vault is easily visualized in the area where the handle of the manipulator is seen. The uterosacral ligaments are also apparent, and the posterior cul-de-sac is not obliterated.

#### 3.1.2. Presence of CCDSO

**Figure 3:**
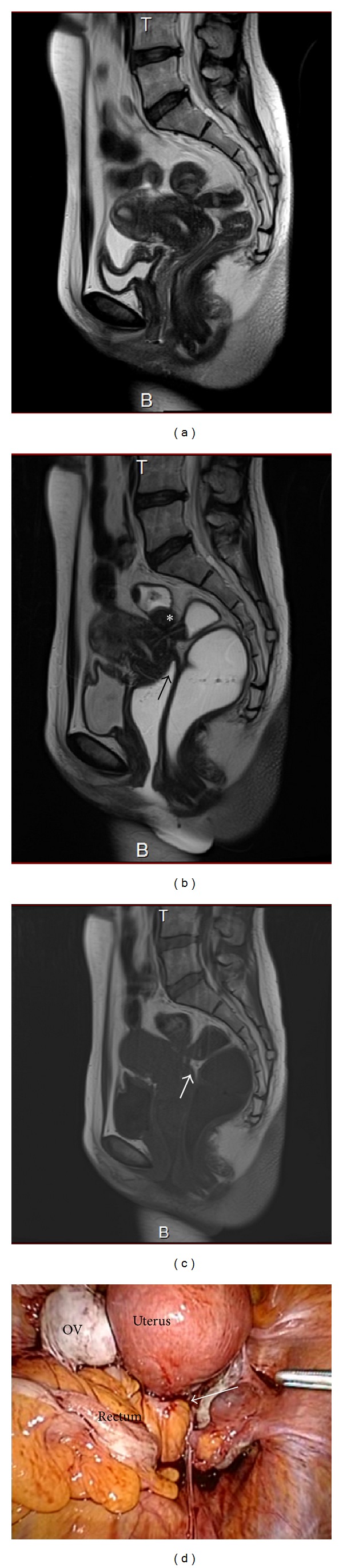
(a) MR image obtained before jelly administration in a patient with ovarian cyst and CCDSO. (b) MR image obtained after jelly administration in the same patient. The posterior vaginal vault is tethered (black arrow). The rectal surface is tethered (asterisk). (c) T1WI from the same patient showing a high-intensity lesion (white arrow). (d) Pelvic laparoscopy findings in the same patient. The ovarian cyst and obliterated Pouch of Douglas are seen (white arrow).

MR images from a case of ovarian cyst and typical CCDSO are shown in Figures 3(a)–3(d).  Figure 3(a) is an MR image obtained before jelly administration, whereas Figure 3(b) is an MR image obtained after jelly administration. The posterior vaginal vault is tethered, and the posterior cul-de-sac is obliterated. The rectal surface is tethered, and a high-intensity lesion is seen on T1WI (Figure 3(c)).  Figure 3(d) is an image obtained upon pelvic laparoscopy in the same patient. The ovarian cyst and Pouch of Douglas obliteration are clearly seen.

## 4. Discussion

Endometriosis is one of the most common diseases encountered in gynecological practice. Patients with endometriosis have various symptoms and conditions, and a wide variety of treatment options are available [8, 9]. CCDSO scores can be as high as 40 or more according to the rASRM scoring system, reaching a severe Stage IV condition [7, 10]. CCDSO can cause various types of pain including menstrual, chronic pelvic, and defecation pain, as well as dyspareunia, and can thereby significantly compromise QOL [9]. To date, the only definitive means of diagnosing endometriosis and assessing its severity, according to criteria such as those of the rASRM scoring system, is direct visualization during abdominal operative procedures, including laparoscopic surgery [7, 10]. Critical organs including the rectum and ureters are adjacent to the Pouch of Douglas, making injury to these structures a concern during adhesiolysis [3]. Therefore, adequate presurgical assessment and understanding of the distribution and extent of endometriosis are essential. CCDSO is not always anticipated presurgically. It can be incidentally encountered during surgery. Thus, a reliable preoperative diagnostic method is needed.

### 4.1. Imaging Modalities Used for Diagnosing CCDSO

Transvaginal US, rectal endoscopic US, MR imaging, and laparoscopy are reportedly used for diagnosing CCDSO, and each approach has its specific advantages [11–23]. MR imaging provides good soft tissue delineation and visualization of the entire pelvis including the support structures [18, 19]. However, nonneoplastic lesions such as adhesions involving the Pouch of Douglas are somewhat difficult to detect on MR images without contrast medium. We have reported the utility of the MR imaging jelly method for diagnosing CCDSO [5, 6]. This imaging technique facilitates the diagnosis of bowel endometriosis. A reported study showed MR imaging at 3.0T to be valuable in the diagnosis of CCDSO because it allows for detailed interpretation of images owing to the high spatial resolution [21]. In our study, MR imaging was performed at 1.5T. A method that is similar to ours has also been reported [22, 23].

### 4.2. Usefulness of the MR Imaging Jelly Method

Our MR imaging jelly method was shown by the present study to be a useful diagnostic approach for deep endometriosis. Comparison of the images obtained with and without jelly administration confirmed its usefulness. The MR imaging jelly method is a convenient approach that requires no special devices, places minimal burden on patients, and is highly cost effective [5, 6]. The US jelly administered in this study is routinely used for US, making the purchase of new materials or devices unnecessary. Moreover, no adverse events occurred either in our previous studies [5, 6] or in the present study. A gynecologist administered the jelly to all 30 patients of our study; however, administration of the jelly is quite easy because only catheter insertion is necessary. No device specific to gynecologic practice is needed. Thus, not only gynecologists but also nurses and medical radiology technicians can easily be trained to administer the jelly.

### 4.3. Limitations

This study has several limitations. First, our study group comprised only 30 subjects. Although statistically significant results were obtained, it is possible that group homogeneity influenced our study results. We do not believe this to be the case, especially because all of the enrolled patients had symptoms such as menstrual pain but not all were found to have CCDSO. In addition, some of the patients enrolled were scheduled to undergo surgery based on a comparison of their intra-abdominal findings with the MR images used to evaluate the accuracy of the jelly method. Second, all of the images were reinterpreted after surgery. As described above, the assessments were performed in a blinded manner and in random order. However, because of the small number of subjects (*n* = 30), we cannot be certain that the radiologists did not recall the specific patients to which the MR images belonged. Third, there may be a concern regarding reproducibility of our diagnostic method at other institutions. Of the two radiologists that interpreted MR images, one is a specialist in the field of gynecologic imaging and was closely involved in development of the radiographic method at our hospital. Thus, the other less experienced radiologist (a fellow) also interpreted the MR images, and the findings of the two radiologists were compared. However, even the less experienced radiologist achieved clinically meaningful results from the MR imaging jelly method, indicating that this method is likely to be useful for diagnosing CCDSO at other institutions.

## 5. Conclusion

The MR imaging jelly method was shown by our single-center study to be a useful, beneficial, and minimally invasive approach for diagnosing CCDSO. We believe that the MR imaging jelly method will be recognized as a superior diagnostic approach by patients suffering from endometriosis, by gynecologists who plan therapeutic strategies and treat these patients, by radiologists performing diagnostic imaging to provide important information for devising treatment strategies, and by all other personnel involved in the care of patients with endometriosis. The jelly method increased the sensitivity and specificity of MR imaging for diagnosis of adhesions in the Pouch of Douglas.
